# Effects of dietary fennel (*Foeniculum vulgare* Mill.) seed powder supplementation on growth performance, nutrient digestibility, small intestinal morphology, and carcass traits of broilers

**DOI:** 10.7717/peerj.10308

**Published:** 2021-01-28

**Authors:** Huihui Liu, Jinlu Li, Shuqin Lin, Ting Liu, Chen Zheng

**Affiliations:** College of Animal Science and Technology, Gansu Agricultural University, Lan Zhou, Gansu, China

**Keywords:** Fennel seed powder, Growth performance, Apparent metabolic rate, Carcass traits, Intestinal morphology

## Abstract

**Background:**

With the increasing demands in livestock and poultry breeding and the growing number of food-borne diseases, it is necessary to practice food safety and develop strategies to produce healthy livestock. Fennel (*Foeniculum vulgare* Mill.) has been used as an additive in poultry production by some researchers, but there are few studies on the systemic beneficial effects of dietary fennel seed powder supplementation on broilers. Therefore, this study aimed to investigate the effect of dietary fennel seed powder supplementation on feed intake, the apparent metabolic rate of nutrients, intestinal morphology, and carcass traits in Cobb broilers.

**Methods:**

A single-factor experimental design was used. In total, 160 1-day-old Cobb broiler chicks were randomly assigned to four treatments, with four replicates each (*n* = 10/replicate). Broilers in the control (CN) group were fed a basal diet without fennel seed powder, and broilers in the treatment groups were fed a basal diet supplemented with 0.15% (LF), 0.30% (MF), or 0.45% (HF) fennel seed powder, respectively. Feeding trials lasted for 42 days under the conditions of ad libitum access to feed and water, and 24-h illumination. During the third and sixth weeks, digestive and metabolic assays were carried out. When the broilers were 42 days old, one chicken with a weight close to the average was selected from each repetition, euthanized by an intravenous injection of 5% sodium pentobarbital, and carcass traits were measured and intestinal samples were collected for morphological assessment.

**Results:**

There was no significant difference in growth performance of broilers (*P* > 0.05). The breast muscle percentage, fat width and fat width index, breast muscle area, and breast muscle area index of broilers in the LF group were higher than those in other groups (*P* < 0.05). Jejunum weight and length were higher in MF than in CN and LF broilers (*P* < 0.05). Additionally, duodenal villi height, ileal villi height, and ileal wall thickness were higher in MF than in CN broilers (*P* < 0.05). There were no significant differences in nutrient utilization among all groups (*P* > 0.05), except that the ash apparent metabolic rate in MF broilers at 21 days of age was higher than that in LF broilers (*P* < 0.05). In conclusion, dietary supplementation with a moderate concentration of fennel affects carcass performance, and intestinal morphology, and promotes the growth and development of broilers.

## Introduction

With the rapid development of livestock and poultry breeding, the number of reported cases of food-associated infections and the occurrence of drug residues, including antibiotics, synthetic drugs, steroids, and other feed additives in meats, eggs, and milk have increased rapidly. Thus, it is very important to focus on food safety from both the consumers’ and the food industries’ perspectives (Kăcániová et al., 2019). The European Union has banned the use of most growth-promoting antibiotics in feed additives due to issues such as the development of cross-resistance against pathogens and residue accumulation in tissues (Ertas et al., 2005; Olgun & Yıldız, 2014; Kăcániová et al., 2019). Accordingly, the search for safe and low-toxicity feed additives has gained increased research interest. Plants, especially herbs, have been used for medicinal purposes for centuries, and some have played a significant role in the maintenance of animal health (AI-Kassie, 2008). Several studies have been conducted on herbal additives owing to their many advantages, including low cost, easy availability, and lack of residual effects, and because of the absence of concerns regarding the use of antibiotics (Saeedi et al., 2017). Supplementation of livestock and poultry feed with plants containing bioactive components has produced encouraging results. These additives can promote growth and performance, and improve feed efficiency, nutrient digestion, antioxidant status, immunological indices, and poultry health (Dhama et al., 2015; Alagawany et al., 2019).

Fennel (*Foeniculum vulgare* Mill.) is a common herbal medicine and a popular aromatic plant that belongs to the family Apiaceae and is related to cumin, dill, caraway, and anise, which all bear aromatic fruits that are commonly called seeds (Dhayalan, Jegadeeshwari & Gandhi, 2015). Fennel’s seed is commonly used as a natural remedy against digestive disorders such as dyspepsia, bloating, and flatulence, and possesses analgesic, antipyretic, and antioxidant properties (El-Deek, Attia & Hannfy, 2003; Choi & Hwang, 2004; Guimarăes et al., 2011). It is also used to flavor foods, liqueurs and in the perfumery industry (Choi & Hwang, 2004). Essential oils derived from fennel have antioxidant, antimicrobial, and hepatoprotective activities. They are composed of several monoterpenes and phenylpropanoids, where trans-anethole, estragole, fenchone and limonene exist as main constituents. trans-anethole, often the most prevalent constituents, counts for the anise taste, fenchone provides the bitterness, and estragole (methylchavicol) the sweetness (Ruberto et al., 2000; El-Deek, Attia & Hannfy, 2003; Oktay, Gülçin & Küfrevioğlu, 2003; Choi & Hwang, 2004; Gharaghani, Shariatmadari & Karimi Torshizi, 2013).

The use of fennel in poultry diets improves appetite, digestion, nutrient absorption, and immunity, and does not produce drug residues. Mohammed & Abbas (2009) demonstrated that broilers fed diets containing various levels of fennel seed showed improved weight gain and feed efficiency. Similarly, Abou-Elkhair, Selim & Hussein (2018) reported that laying hens fed a diet supplemented with fennel, black cumin tended to have a higher body weight gain than control diet. El-Deek, Attia & Hannfy (2003) reported significantly improved color intensity when a mixture of 0.1% of fennel plus anise was supplemented to broiler diets. Therefore, in this study, we hypothesized that fennel seed powder (FSP) would improve growth performance, carcass performance, nutrient utilization, and intestinal morphology in broilers. To test this hypothesis, we investigated the effects of dietary supplementation with different concentrations of FSP on these features.

## Materials and Methods

### Ethics statement

The study protocol was reviewed and approved by the Animal Welfare Ethics Committee of Gansu Agricultural University (Approval No. DK-005). And the animal procedures used in this study strictly abide by the Administrative Measures of Gansu Province on Experimental Animals (2005–2012).

### Experimental animals and design

In total, 160 1-day-old healthy, male Cobb broiler chicks were obtained from Baoji Dacheng Poultry Co., Ltd. (Shanxi, China). The chicks were randomly assigned to four treatments, with four replicates each (*n* = 10/replicate). A single-factor experimental design was used. The treatments were as follows: different basal diets were fed during the initial (0–21 days) and finisher (21–42 days) stages (Table 1), and broilers in the control (CN) group were fed a basal diet without FSP, whereas those in the treatment groups were fed a basal diet supplemented with 0.15% (LF), 0.30% (MF), or 0.45% (HF) FSP. Fennel seeds were purchased from the local market (Gansu, China). The basal diets, based on corn and soybean meal, were formulated according to the nutritional requirements of broilers in China (2004) (Table 1).

**Table 1:** Ingredients and nutrient composition of basal diets.

Items	Initial (1–21 days)	Finisher (22–42 days)
Ingredients (%)		
Corn	46.84	55.91
Bran	6.24	1.12
Soybean meal	31.05	28.00
Cottonseed meal	6.41	6.35
Rapeseed meal	5.60	5.20
Calcium phosphate	1.32	1.17
Limestone powder	1.22	1.10
Methionine	0.15	0.07
Salt	0.27	0.18
50% Choline chloride	0.40	0.40
Additive premix^1^	0.50	0.50
Total	100	100
Nutrient composition^2^		
Metabolizable energy (kcal/kg)	12.54	12.96
Crude protein (%)	21.49	20.00
Crude fiber (%)	3.65	4.24
Ether extract (%)	3.42	3.12
Lysine (%)	1.00	1.07
Methionine (%)	0.45	0.40
Calcium (%)	1.19	0.90
Available phosphorus (%)	0.50	0.41
Sodium (%)	0.20	0.30
Chloride (%)	0.18	0.15

**Notes:**

1 Additive premixes were available/kg at 1–3 weeks/4–6 weeks: Fe, 120/80 mg (ferrous sulfate); Zn, 120/80 mg (zinc sulfate); Cu, 10/8 mg (copper sulfate); Mn, 120/100 mg (manganese sulfate); I, 0.70 mg (calcium iodate); Se, 0.30 mg (sodium selenite); vitamin A, 2700 IU; vitamin D, 3400 IU; vitamin E, 10 IU; vitamin K, 0.5 mg; thiamin, 1.8 mg; riboflavin, 7.2 mg; pantothenic acid, 10 mg; niacin, 27 mg; pyridoxine, 3 mg; biotin, 0.15 mg; folic acid, 0.55 mg; vitamin B_12_, 0.009 mg.

2 Crude protein, calcium, phosphorus, ether extract, and ash were analyzed. Metabolizable energy and amino acid content were calculated.

Each chick was numbered according to the treatment. In the first week of the experiment, Multi-Vitamins (Shanghai Neotrieon Boi-Tech Co., Ltd, Shanghai China) were added in the drinking water to relieve stress. The chicks used a vacuum drinker in the first 3 weeks, and in the fourth week, the vacuum drinker was replaced with a nipple drinker. Feed and water were provided ad libitum. The chicks were housed under a combination of natural and artificial auxiliary light, and the illumination time varied depending on the weather and temperature. The temperature and humidity were adjusted to the needs in different growth stages based on daily monitoring of the behavior of the chickens. The room temperatures for broiler were 34–32, 32–28, 28–26, 26–24, 24–22, and 24–22 °C for the first, second, third, fourth, fifth, and sixth week, respectively. Chicks were vaccinated against Newcastle disease (Yangling Green Bio-engineering Co., Ltd, Shanxi, China) at 7 days and 21 days of age, and against mucosal sac disease (Yangling Green Bio-engineering Co., Ltd, Shanxi, China) at 15 days and 28 days of age.

### Measurements

#### Growth performance measurements

Body weight (BW) and feed intake were measured for each replication group each week. Then, the growth performance of broilers was calculated by the average daily feed intake (ADFI), average daily gain (ADG) and ratio of feed to gain (F/G).

#### Nutrient digestibility measurements

Digestive and metabolic assays were carried out from days 18 to 21 and days 39 to 42; starting from 7:00 am, feces were collected. Fecal samples from the same repeat group were mixed, and a certain fraction of the pooled sample was placed in an aluminum box, sprayed with 10 mL of 10% sulfuric acid solution per 100 g of sample, and dried in an oven at 65 °C. In addition, during days 18 to 21 and days 39 to 42, 20–30 g dietary samples were collected daily, mixed, and transported to the laboratory. Dietary and fecal samples were pulverized using a 0.45-mm sieve for nutrient composition analysis of the following feed constituents according to guidelines of the Association of Official Analytical Chemists methods (AOAC, 2002): analytical crude protein (CP; method 990.03), crude ash (Ash; method 942.05), dry matter (DM; method 930.15), and organic matter (OM).

#### Carcass and intestinal measurements

At 42 days of age, one chick that had a body weight close to the average was selected from each replicate group. All chicks were treated humanely and with efforts to minimize suffering. To induce loss of consciousness and death with a minimum of pain and distress, chicks were euthanized by an intravenous injection of 5% sodium pentobarbital (Yin et al., 2018). No criteria were established for euthanizing animals prior to the planned end of the experiment, euthanasia was carried out only to alleviate suffering. Immediately following euthanasia, the abdominal cavity was opened, the pancreas, liver and small intestines were removed, and to determine carcass characteristics. The carcass weight, half-eviscerated weight, eviscerated weight, abdominal fat weight, breast muscle weight, breast muscle area, fat width, and leg muscle weight were recorded, and percentages were calculated (Attia et al., 2015). Simultaneously, the small intestines were segmented and measured. The length and weight of the duodenum, jejunum, ileum, and cecum were measured, and the intestinal index was calculated for the same (Ding & Zhang, 2009). The following formulas were used:

Carcass percentage (%) = carcass weight/live weight × 100;
Half-eviscerated percentage (%) = half-eviscerated weight/live weight × 100;
Eviscerated percentage (%) = eviscerated weight/live weight × 100;
Abdominal fat percentage (%) = abdominal fat weight/eviscerated weight × 100;
Breast muscle percentage (%) = breast muscle weight/eviscerated weight × 100;
Leg muscle percentage = leg muscle weight/eviscerated weight × 100;
Fat width index = fat width (mm)/live weight (kg);
Breast muscle area index = breast muscle area (cm^2^)/live weight (kg);
Intestinal weight index = intestinal weight (g)/live weight (kg);
Intestinal length index = intestinal length (cm)/live weight (kg);

The remaining broiler chickens that survived in the slaughter experiment were sold to local farms and farmers at the end of the experiment to achieve a certain economic value.

#### Intestinal histology morphology measurement

Tissue blocks of approximately one cm^2^ were collected from the middle part of the duodenum, jejunum, and ileum and rinsed gently with physiological saline and then, were fixed in paraformaldehyde solution. The intestine tissue was transversely trimmed, and the sections of five microns were stained with hematoxylin and eosin. Sectioned for morphological analysis using a DP71 microscope (Olympus Corporation, Ltd, Tokyo, Japan) equipped with a camera. Image-Pro Express 6.0 image processing software (Media Cybernetics, Inc., Rockville, MD, USA) was used to measure villus height (from the tip of the villus to the villus-crypt junction), crypt depth (from villus-crypt junction to the base of the crypt), and intestinal wallthickness of the duodenum, jejunum, and ileum. Four representative visual fields were selected in each section, and the above features were measured thrice in each visual field, and the results were averaged (Liu et al., 2020; Zhang et al., 2020).

### Evaluation of meat quality indicators

The pressure loss method (Farouk, Wieliczko & Merts, 2004) was used to measure the water loss rate. Approximately 1.5 g of each breast and leg muscle samples devoid of fat, tendons, and sarcolemma were compressed to a 5 cm × 5 cm × 1 cm thin piece of meat along the vertical direction of the muscle fibers. Weight before and after compression were determined.

The tenderness of breast muscle was measured using TA-XTM tenderness tester (Bosin Industrial Development Co., Ltd., Shanghai, China).

Within 1 h after slaughter, breast muscle samples of approximately 3 cm width and 2 cm thickness were weighed (W1). Then, the meat samples were placed inside a plastic bag without contacting the wall of the bag. The bags were sealed and stored at 4 °C for 24 h, and then weighed (W2).

Breast muscle drip loss (%) = W2/W1 × 100.

Within 1 h after slaughter, approximately 100 g of breast muscle was collected and weighed (W1). The samples were placed in a steamer for 30 min, moved to a cool place, and weighed again after 20 min (W2).

Cooked meat rate (%) = W2/W1 × 100.

To measure pH, breast muscle and leg muscle samples were placed in glass dishes, and an acidity meter was inserted directly into the meat at three different time points (15 min, 1 h, and 24 h).

### Statistical analysis

Data were analyzed by one-way ANOVA (SPSS 19.0, IBM, Chicago, IL, USA) using the following equation:

*X*_*ij*_ = μ + α_*i*_ + *e*_*ij*_,

where *X*_*ij*_ is the observation of the dependent variable (*i*, 1–4, *j*, 1–4), μ is the population mean, α_*i*_ is the *i*th treatment effect, and *e*_*ij*_ is the random error associated with the observation. Significance was defined as *P* ≤ 0.05, and a tendency as 0.05 < *P* ≤ 0.20, using Tukey’s multiple comparison test.

## Results

### Effect of FSP on growth performance of broilers

Table 2 shows the effects of the supplementation of FSP in broiler diets on growth performance. Based on the obtained results, the addition of different levels of FSP in the diet of broilers had no significant differences in BW, ADG, ADFI and F/G.

**Table 2:** Effects of dietary fennel seed powder supplementation at three levels on body weight (g), average daily gain (g), average daily feed intake (g), and feed-to-weight ratio (%) in broilers.

Parameter^1^	CN^2^	LF	MF	HF	*P* value
BW	2,111.38 ± 128.34	2,118.00 ± 149.84	2,080.94 ± 160.28	2,098.48 ± 209.94	0.587
ADG	49.45 ± 3.08	49.42 ± 3.62	48.55 ± 3.78	48.97 ± 4.97	0.986
ADFI	96.41 ± 9.09	94.68 ± 5.82	96.83 ± 3.76	97.41 ± 5.57	0.937
F/G	1.96 ± 0.28	1.92 ± 0.17	2.00 ± 0.16	2.00 ± 0.21	0.939

**Notes:**

1 BW, body weight; ADG, average daily gain; ADFI, average daily feed intake; F/G, ratio of feed to gain.

2 CN, basal diet without FSP; LF, 0.15% FSP; MF, 0.30% FSP; HF, 0.45%.

### Effect of FSP on apparent nutrient digestibility in broilers

Table 3 presents the nutrient digestibility affected by the additive of FSP in broiler diets. The results indicate that the apparent digestibility of DM, OM, and CP in broilers were not significantly different among the different levels of FSP supplementation (*P* > 0.05), except for the apparent digestibility of Ash in LF broilers was significantly lower than that in MF broilers (*P* < 0.05).

**Table 3:** Effect of dietary fennel seed powder supplementation at three levels on the apparent metabolic rate in broilers (%).

Items	CN^1^	LF	MF	HF	*P* value^2^
21 days					
DM	72.96 ± 2.94	71.15 ± 3.25	74.49 ± 3.58	70.3 ± 1.67	0.238
OM	75.29 ± 2.68	74.11 ± 3.10	76.63 ± 3.27	72.68 ± 1.40	0.254
CP	63.24 ± 7.42	67.22 ± 3.30	68.02 ± 4.10	64.3 ± 4.10	0.710
Ash	38.56 ± 6.87ab	27.46 ± 5.71b	42.79 ± 8.40a	35.04 ± 5.84ab	0.044
42 days					
DM	65.95 ± 3.22	68.92 ± 2.25	68.78 ± 1.15	70.61 ± 5.35	0.315
OM	68.90 ± 3.08	71.36 ± 2.27	71.71 ± 1.10	73.13 ± 5.02	0.346
CP	48.76 ± 2.76	51.91 ± 9.02	48.56 ± 7.02	53.55 ± 9.76	0.749
Ash	19.36 ± 6.96	30.17 ± 10.92	22.17 ± 3.55	30.51 ± 11.47	0.233

**Notes:**

1 CN, basal diet without FSP; LF, 0.15% FSP; MF, 0.30% FSP; HF, 0.45% FSP.

2 Means within a row lacking a common superscript differ (*P* < 0.05).

### Effect of FSP on carcass traits of broilers

The results of carcass traits are shown in Table 4.The breast muscle percentage was significantly affected by levels of FSP (*P* < 0.05),and decreased with increasing FSP. Supplemented with LF group FSP had significantly increased fat width and the fat width index than in the other three groups (*P* < 0.05). The breast muscle area was higher in LF than in CN and HF broilers (*P* < 0.05), and breast muscle area index was higher in LF than in HF broilers (*P* < 0.05).

**Table 4:** Effect of dietary fennel seed powder supplementation at three levels on carcass traits of broilers.

Parameter^1^	CN^2^	LF	MF		HF	*P* value^3^
live weight (kg)	2.08 ± 0.12	2.17 ± 0.15	2.18 ± 0.16	2.13 ± 0.15		0.769
CW (kg)	1.94 ± 0.14	2.04 ± 0.16	2.03 ± 0.16	2.01 ± 0.14		0.796
CP (%)	93.27± 2.32	94.07 ± 1.93	93.32 ± 2.32	94.76 ± 0.38		0.980
HEW (kg)	1.80 ± 0.12	1.89 ± 0.05	1.88 ± 0.95	1.85 ± 0.12		0.568
HEP (%)	86.59 ± 2.33	87.68 ± 1.32	86.41 ± 1.48	86.84 ± 1.85		0.899
EW(kg)	1.56 ± 0.10	1.65 ± 0.07	1.64 ± 0.06	1.60 ± 0.08		0.431
EP (%)	75.19 ± 2.37	76.36 ± 1.53	75.62 ± 3.87	75.09 ± 1.36		0.983
AFW (g)	23.05 ± 9.83	17.98 ± 6.43	21.13 ± 7.17	24.76 ± 4.17		0.596
AFP (%)	1.48 ± 0.67	1.09 ± 0.38	1.29 ± 0.45	1.54 ± 0.24		0.518
BMW (kg)	0.54 ± 0.04	0.62 ± 0.07	0.54 ± 0.08	0.52 ± 0.04		0.141
BMP (%)	34.33 ± 1.28ab	37.77 ± 2.23a	32.97 ± 3.85b	32.65 ± 0.99b		0.045
LMW (kg)	0.25 ± 0.03	0.26 ± 0.02	0.22 ± 0.03	0.26 ± 0.04		0.306
LMP (%)	16.24 ± 1.38ab	15.83 ± 0.70ab	13.58 ± 1.20b	16.47 ± 1.66a		0.040
Fat width (mm)	0.72 ± 0.02b	0.93 ± 0.04a	0.71 ± 0.01b	0.74 ± 0.01b		0.022
FWI (mm/kg)	0.35 ± 0.025b	0.43 ± 0.025a	0.33 ± 0.025b	0.35 ± 0.02b		0.011
BMA (cm^2^)	65.13 ± 1.38a	65.75 ± 3.38a	59.88 ± 4.50ab	56.38 ± 4.99b		0.013
BMAI (cm^2^/kg)	31.39 ± 1.98ab	30.39 ± 1.56a	27.49 ± 1.82ab	26.35 ± 0.73b		0.002

**Notes:**

1 CW, carcass weight; CP, carcass percentage; HEW, half-eviscerated weight; HEP, half-eviscerated percentage; EW, eviscerated weight; EP, eviscerated percentage; AFW, abdominal fat weight; AFP, abdominal fat percentage; BMW_,_ breast muscle weight; BMP_,_ breast muscle percentage; LMW, leg muscle weight; LMP, leg muscle percentage; FWI, fat width index; BMA, breast muscle area; BMAI, breast muscle area index.

2 CN, basal diet without FSP; LF, 0.15% FSP; MF, 0.30% FSP; HF, 0.45% FSP.

3 Means within a row lacking a common superscript differ (*P* < 0.05).

### Effect of FSP on digestive organs, intestinal development, and morphology of broilers

Jejunum length and weight were significantly higher in the MF group than in the other groups (*P* < 0.05), and did not significantly differ among the other groups (*P* > 0.05) (Table 5).

**Table 5:** Effects of dietary fennel seed powder supplementation at three levels on digestive organ weight (g) and length (cm) in broilers.

Parameter	CN^1^	LF	MF	HF	*P* value^2^
Stomach weight					
Muscular stomach	15.74 ± 1.16	16.67 ± 2.37	18.26 ± 2.05	18.57 ± 3.25	0.315
Glandular stomach	5.46 ± 0.48	5.98 ± 0.96	6.03 ± 0.68	5.45 ± 0.82	0.565
Intestinal segment weight					
Duodenum	10.88 ± 2.04	10.21 ± 1.45	12.36 ± 0.71	10.00 ± 1.59	0.175
Jejunum	18.54 ± 1.31b	20.50 ± 1.67b	23.67 ± 2.26a	18.27 ± 1.54b	0.030
Ileum	14.92 ± 1.16	15.67 ± 0.61	18.05 ± 2.75	15.70 ± 1.64	0.113
Cecum	15.25 ± 1.18	14.50 ± 1.64	15.50 ± 1.42	15.00 ± 0.81	0.733
Intestinal segment length					
Duodenum	12.25 ± 0.91	11.25 ± 0.92	13.00 ± 1.73	10.75 ± 0.35	0.369
Jejunum	50.75 ± 3.32b	52.50 ± 4.87b	62.25 ± 4.09a	57.02 ± 4.45ab	0.010
Ileum	52.50 ± 4.03	50.00 ± 3.71	54.75 ± 3.25	54.50 ± 2.69	0.236
Cecum	15.25 ± 0.89	14.50 ± 1.32	15.50 ± 1.45	15.00 ± 1.24	0.708

**Notes:**

1 CN, basal diet without FSP; LF, 0.15% FSP; MF, 0.30% FSP; HF, 0.45% FSP.

2 Means within a row lacking a common superscript differ (*P* < 0.05).

Table 6 shows the impact of different funnel supplementation on intestinal weight and length index. The highest intestinal weight index of duodenum, jejunum and ileum recorded in the MF group (*P* > 0.05). The highest intestinal length index of jejunum, ileum and total intestinal were observed in the MF group (*P* > 0.05).These results reflect the positive effect of feeding the broilers with a diet supplementation 0.3% FSP on the intestinal development.

**Table 6:** Effects of dietary fennel seed powder supplementation at three levels on intestinal weight index and length index (%) of broilers.

Parameter	CN^1^	LF	MF	HF	*P* value^2^
Intestinal weight index					
Duodenum	5.25 ± 1.29a	4.70 ± 1.12b	5.70 ± 1.12a	4.67 ± 0.92b	0.035
Jejunum	8.94 ± 0.82b	9.51 ± 1.26b	10.84 ± 0.47a	8.55 ± 0.25b	0.018
Ileum	7.19 ± 0.74b	7.25 ± 0.38b	8.25 ± 0.92a	7.37 ± 0.95ab	0.033
Cecum	7.35 ± 0.67	6.71 ± 0.82	7.10 ± 0.24	7.04 ± 0.62	0.567
Intestinal length index					
Duodenum	5.90 ± 0.45	5.20 ± 0.47	6.00 ± 0.97	5.04 ± 0.29	0.101
Jejunum	24.45 ± 1.92b	24.28 ± 2.36b	28.76 ± 3.97a	26.70 ± 1.49ab	0.098
Ileum	25.32 ± 2.7ab	23.13 ± 2.06b	25.15 ± 1.23a	25.56 ± 1.50ab	0.016
Cecum	7.36 ± 0.76	6.70 ± 0.64	7.10 ± 0.17	7.02 ± 0.49	0.458
Total intestinal	146.00 ± 5.25b	142.37 ± 16.56b	167.50 ± 9.24a	153.00 ± 14.76ab	0.047

**Notes:**

1 CN, basal diet without FSP; LF, 0.15% FSP; MF, 0.30% FSP; HF, 0.45% FSP.

2 Means within a row lacking a common superscript differ (*P* < 0.05).

**Figure 1:**
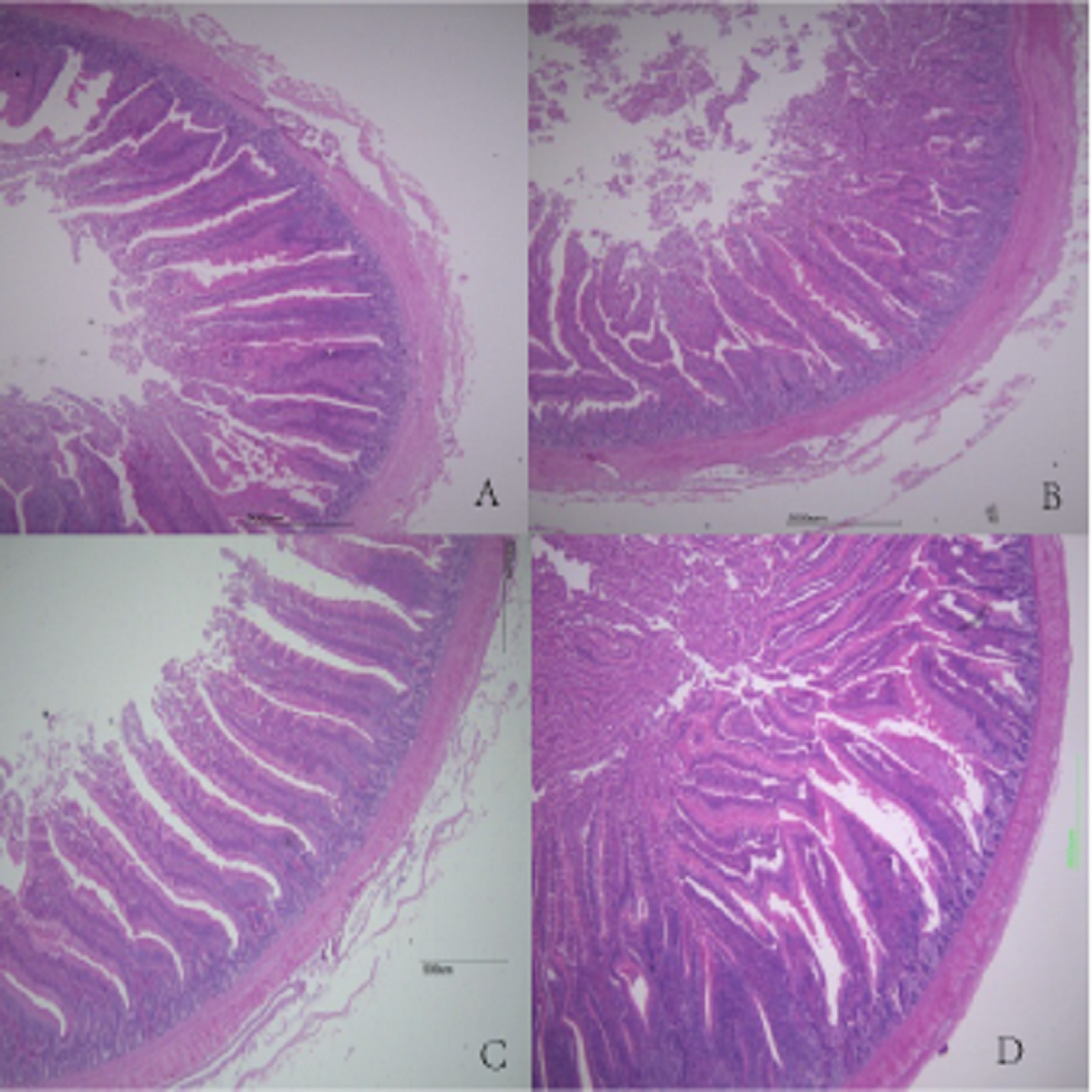
Histological figures of duodenum. (A) Basal diet without FSP; (B) 0.15% FSP; (C) 0.5% FSP; (D) 0.45% FSP.

**Figure 2:**
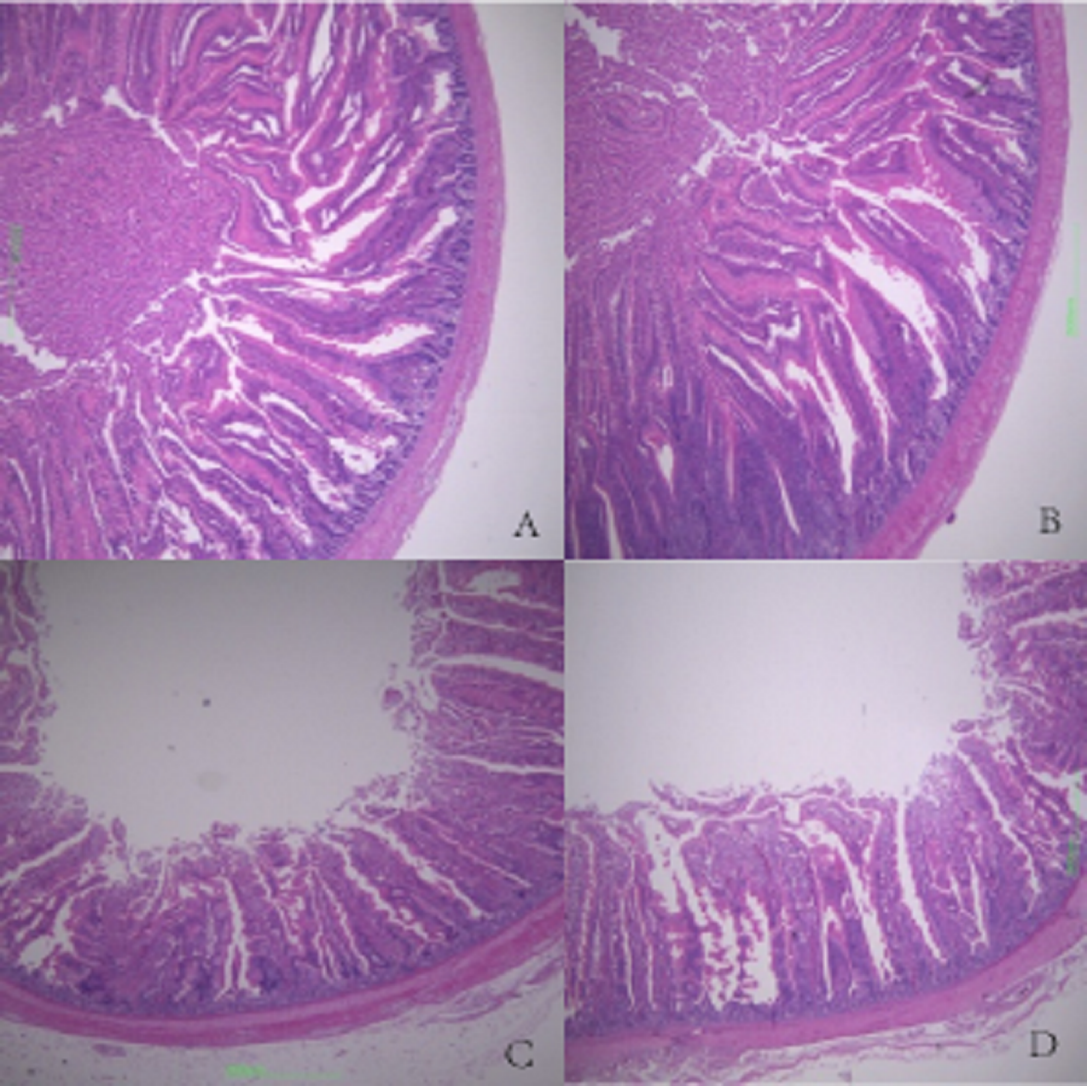
Histological figures of jejunum. (A) Basal diet without FSP; (B) 0.15% FSP; (C) 0.5% FSP; (D) 0.45% FSP.

**Figure 3:**
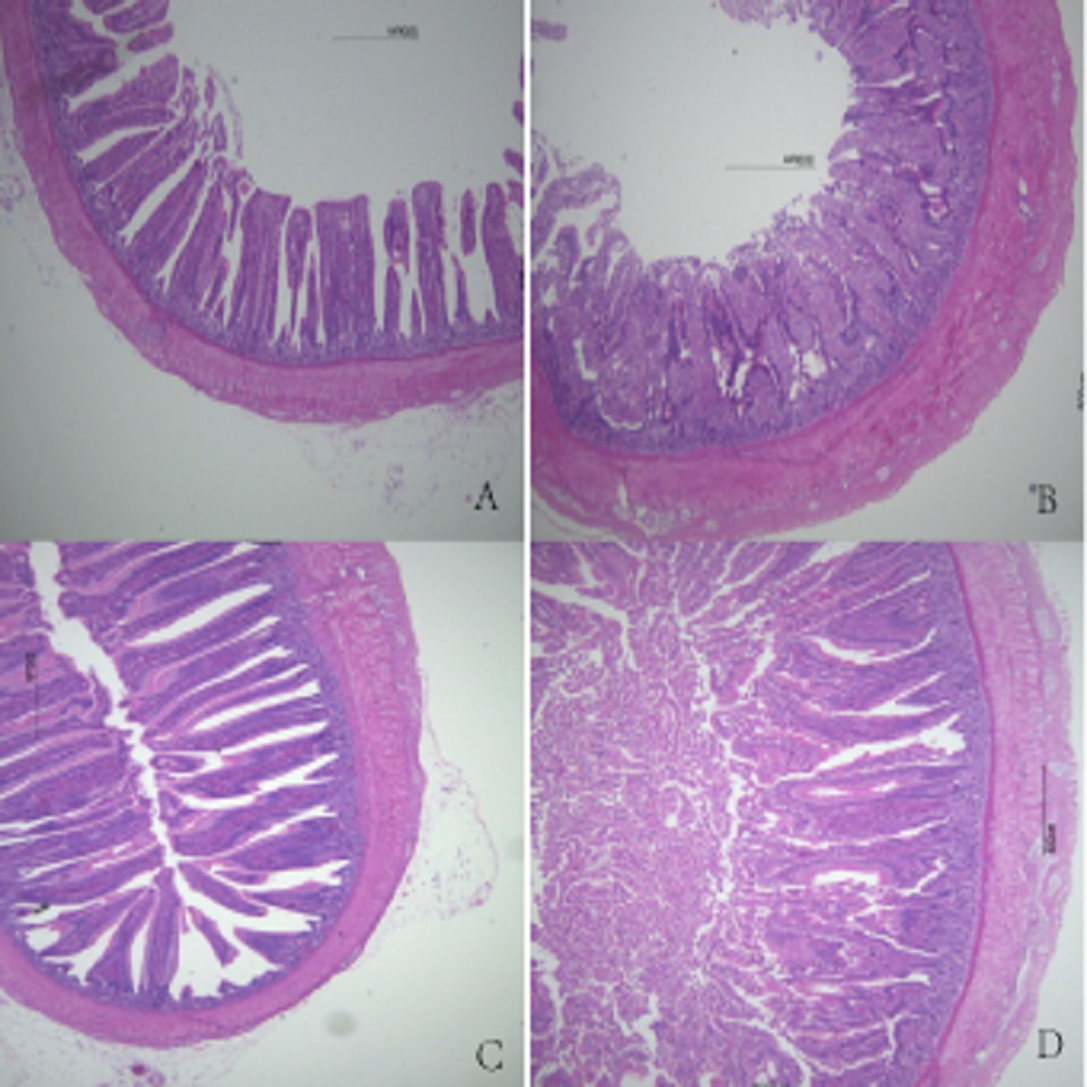
Histological figures of ileum. (A) Basal diet without FSP; (B) 0.15% FSP; (C) 0.5% FSP; (D) 0.45% FSP.

Duodenal villus height, ileal wall thickness, and ileal villus height were significantly higher in MF broilers than in the other groups (*P* < 0.05). There were no significant differences in the other features evaluated among the other groups (*P* > 0.05). There was a gradual increase in intestinal wall thickness, crypt depth, and villus height in duodenum, jejunum, and ileum with increasing FSP level (*P* > 0.05), except for HF group (Table 7). Histological figures of duodenum, jejunum and ileum are shown in Figs. 1–3.

**Table 7:** Effect of dietary fennel seed powder supplementation at three levels on intestinal morphological index (µm) of broilers.

Parameter	CN^1^	LF	MF	HF	*P* value^2^
Intestinal wall thickness					
Duodenum	238.22 ± 29.89	260.52 ± 33.05	280.06 ± 44.23	278.55 ± 36.19	0.364
Jejunum	252.56 ± 53.91	262.73 ± 33.94	309.35 ± 48.08	284.26 ± 43.05	0.341
Ileum	335.25 ± 47.55b	410.41 ± 75.83ab	453.74 ±55.53a	420.22 ± 58.66ab	0.089
Crypt depth					
Duodenum	209.83 ± 41.45	226.87 ± 29.76	263.72 ± 40.33	245.52 ± 35.37	0.245
Jejunum	202.35 ± 38.91	225.50 ± 28.87	240.15 ± 34.28	226.12 ± 24.67	0.448
Ileum	281.52 ± 50.17	296.08 ± 68.33	320.36 ± 47.14	306.01 ± 52.67	0.789
Villus height					
Duodenum	1314.93 ± 266.30b	1378.09± 294.33ab	1507.44 ± 280.85a	1477.66 ± 363.67ab	0.332
Jejunum	1366.21 ± 193.91	1391.82 ± 141.86	1530.84 ± 236.66	1438.63 ± 110.52	0.588
Ileum	1128.76 ± 159.24b	1211.87 ± 210.67ab	1492.33 ± 220.92a	1369.88 ± 98.82ab	0.058

**Notes:**

1 CN, basal diet without FSP; LF, 0.15% FSP; MF, 0.30% FSP; HF, 0.45% FSP.

2 Means within a row lacking a common superscript differ (*P* < 0.05).

### Effect of FSP on broiler meat quality

There were no significant differences in the meat quality parameters measured among any of the groups (*P* > 0.05). The water loss rate in breast muscle increased with increasing level of FSP in the LF and MF groups; however, it decreased again in the HF group. The water loss rate in leg muscle decreased with increasing levels of FSP in the LF and MF groups; however, it increased in the HF group. Breast muscle drip loss and water loss rate were similar in all groups. Breast muscle tenderness and the cooked meat rate were lower in all experimental groups than in the control group. The pH of breast and leg muscles was higher in all experimental groups than in the control group (Table 8).

**Table 8:** Effect of dietary fennel seed powder supplementation at three levels on the meat quality of broilers.

Parameter		CN^1^	LF	MF	HF	*P* value
Water loss rate (%)						
breast muscle	19.62 ± 4.24		22.87 ± 5.87	23.72 ± 6.71	19.56 ± 3.90	0.588
leg muscle	25.12 ± 3.72		23.23 ± 5.24	20.99 ± 2.72	24.17 ± 3.97	0.416
Breast muscle drip loss (%)	12.61 ± 0.43		12.63 ± 0.51	12.65 ± 0.53	12.57 ± 0.53	0.996
Breast muscle tenderness (kg·f)	3.78 ± 0.88		3.09 ± 0.87	3.34 ± 0.47	2.49 ± 0.57	0.138
Cooked meat rate (%)	69.50 ± 1.48		68.36 ± 1.66	68.18 ± 0.99	69.17 ± 1.10	0.470
pH_45min_						
breast muscle	5.84 ± 0.54		6.36 ± 0.39	6.00 ± 0.32	6.035 ± 0.23	0.323
leg muscle	5.64 ± 0.42		6.16 ± 0.39	5.82 ± 0.21	6.05 ± 0.06	0.131
Abdominal fat percentage (%)	1.47 ± 0.44		1.23 ± 0.36	1.28 ± 0.21	1.54 ± 0.21	0.521

**Note:**

1 CN, basal diet without FSP; LF, 0.15% FSP; MF, 0.30% FSP; HF, 0.45% FSP.

## Discussion

Mohammed & Abbas (2009) reported that the addition of fennel at 1, 2, or 3 g/kg to the feed resulted in significant improvements in body weight and feed efficiency in chickens. Fennel has a specific flavor and can be used as a natural flavoring agent to improve the palatability of feeds, thereby increasing food intake and promoting nutrient absorption in animals (Gende et al., 2009; Wu et al., 2019). In this study, we did not observe significant effects of fennel on weight gain, feed intake, and feed conversion efficiency, the best BW, ADG and ADFI were obtained when broilers were supplemented with 0.15% of FSP In line with our findings, Safaei-Cherehh et al. (2018) observed that diet supplementation with fennel extract lead to feed intake restriction (FIR) of 4.66–7.63%, and corresponding weight reduction (WR) of 4.07–7.33%. AL-Sagan et al. (2020) reported that the addition of 1.6 and 3.2% FSP to the feed did not affect broiler body weight gain, feed intake under normal temperatures, while, under chronic heat stress, results showed significant alterations in broiler performance, production. Moreover, our findings were in agreement with those of (El-Deek, Attia & Hannfy, 2003; Gharaghani, Shariatmadari & Karimi Torshizi, 2013; Abou-Elkhair, Selim & Hussein, 2018). The contrasting effects of fennel on growth performance in various studies could be attributed to differences temperature of room, type of basal diet, concentration of fennel, or treatment methods. Due to the small number of experimental samples and limited levels of FSP supplementation in this study, the effect of FSP on growth performance in broilers needs to be studied further.

In 21-day-old broilers, the digestibility of DM, OM, CP, and Ash was the highest for 0.30% FSP supplementation and decreased at higher and lower concentrations. The results were in line with those reported by Cheng & Xu (2019). This suggested that supplementation of FSP at a medium concentration can improve nutrient metabolism. However, at 42 days, the apparent digestibility of DM, OM, CP, and Ash tended to increase in broilers that were fed 0.45% FSP, indicating a dose-dependent effect of FSP with the increase of broiler age. Lack of research on the effect of fennel on nutrient utilization in broiler chickens, but studies on anise and cumin related to fennel have been reported. Ertas et al. (2005) demonstrated that adding an essential oil mix derived from oregano, clove, and anise to animal feed positively affected digestibility of nutrient. The effect of fennel on improving nutrient utilization was found to be closely related to its role in stimulating appetite, gastric juice secretion, and intestinal peristalsis, and it was not as significant as that of essential oil in broilers, which could be attributed to the fact that essential oil is richer in aromatic components and thus has a more pronounced characteristic flavor. Further, improvement of the nutrient utilization rate in broilers is closely related to improvement of intestinal development. Fennel may stimulate digestive enzyme secretion and activity and is a potential effector of microbial communities (Dorman & Deans, 2000; Platel & Srinivasan, 2000; Williams & Losa, 2001; Burt, 2004), which can further improve nutrient metabolism. Amad et al. (2011) showed that adding 150, 750, or 1,500 mg/kg mixed essential oils of anise and thyme to the feed significantly improved the apparent CP utilization rate in broiler chickens in a dose-dependent manner. Cho, Kim & Kim (2014) reported that adding star anise and thyme essential oils to the feed significantly improved the DM utilization rate and gross energy in broilers. Thus, we speculate that when fennel is combined with other dietary supplements, an additive or synergistic effect may be achieved.

Carcass characteristic is an important economic index in broiler production, and a valuable parameter to measure the net meat production capacity of broilers and to evaluate the meat production performance and feeding effect in animals. Carcass and eviscerated percentages are the main indices to measure the meat production performance of livestock and poultry. It is generally considered that the carcass percentage is above 80%, the eviscerated percentage is above 60%, and the meat performance is good (Li et al., 2019). In this study, the carcass percentage of broiler chickens was 93.86%, and the eviscerated percentage was 75.57%, indicating that the performance of meat was better. Dietary FSP supplementation did not seem to substantially affect carcass characteristics, except for fat width, breast muscle percentage, breast muscle area and fat width index, breast muscle area index were significantly higher in the 0.15% supplementation group. The results of carcass characteristics in this study were in line with those obtained by AL-Sagan et al. (2020), who reported that the supplementation of 1.6% and 3.2% FSP did not show differences for carcass characteristics. Similar results were also reported by Mohammed & Abbas (2009) and El-Deek, Attia & Hannfy (2003).

In this study, the weight and length of the jejunum, the jejunal and ileal weight indices, and the ileal and total intestinal length indices were significantly higher in the 0.30% FSP supplementation group than in the other groups. This suggested that FSP promotes intestinal growth and development in broilers, which could be attributed to the improvement of the intestinal environment by the FSP. Nutrient digestion, absorption, and utilization mainly occur in the small intestine. Good intestinal morphology is essential for digestive function and to promote body growth and development. Intestinal villus height and crypt depth reflect the degree of development and function of the intestinal epithelium (Hampson, 1986). With increasing intestinal villus height, digestion and absorption improve, the incidence of diarrhea decreases, and growth and development accelerate. Shallower crypts indicate an increased rate of cell maturation and increased secretion (Beers-Schreurs et al., 1998). Reisinger et al. (2011) showed that dietary supplementation of essential oils of star anise, oregano, and citrus fruit significantly increased the recess depth and villus height in the ilea of broilers. Hong et al. (2012) found that adding star anise and oregano to the feed significantly increased duodenal villus height in broilers. In our study, when 0.30% FSP was added, duodenal villus height, ileal wall thickness, and ileal villus height were significantly increased. With increasing FSP supplementation level, the wall thickness and villus height in the jejunum and ileum gradually increased. There was no significant difference in crypt depth among the groups. This indicated that FSP supplementation promoted intestinal growth and development and improved intestinal morphology in broilers. Anise has been reported to improve the intestinal microbiota structure (Charal, 2014; Wang et al., 2015), and a similar effect may account for the effects of fennel on intestinal development and morphology, that is, certain active ingredients or unknown factors in fennel promote the growth of beneficial intestinal microorganisms in broilers. A healthy intestinal micro-ecosystem promotes normal intestinal tract development and ensures the integrity of the intestinal morphological structure. However, fennel showed different effects on different intestinal segments in broilers, which could be attributed to differences in the morphology and structure of each intestinal segment or the incomplete utilization of fennel components. This further affected the growth performance of broilers. Further research is needed to back up these findings.

The water-holding capacity of meat tissue is the ability of muscle tissue to retain water, and it is an important index to evaluate the quality of poultry meat. At present, water loss rate, drip loss and cooked meat rate are used to evaluate (Yue et al., 2015). In the present investigation, there is no differences were observed in the meat quality, the FSP-supplemented groups reduced breast muscle tenderness, which seemed to improve the edible quality. The pH of high-quality chicken meat is 6.0–6.5 (Hu, Zhu & Li, 2005). Except for the low pH in leg muscles in the MF group, the pH in LF and HF groups were in the normal range and higher than that in the control group. Too high a pH is harmful to the conversion of normal muscle to edible meat, and too low a pH will cause meat deterioration. Meat pH is related to the water-holding capacity (Brewer, 2004; Jung et al., 2010), meat with a high pH value has a higher water-holding capacity (Hu, Zhu & Li, 2005), which affects the texture, juiciness, and flavor of meat (AL-Sagan et al., 2020).

## Conclusions

In conclusion, dietary supplementation of FSP, resulted in a beneficial impact on broiler digestion and absorption abilities, carcass traits and intestinal histological, especially at a concentration of 0.30%, which improved intestinal morphological development and thus promoting healthy and efficient development in Cobb broiler chickens. But more investigation is needed to determine the optimum concentration to be added to the poultry diets.

## Supplemental Information

10.7717/peerj.10308/supp-1 Supplemental Information 1 Nutrient digestibility.

10.7717/peerj.10308/supp-2 Supplemental Information 2 Body weight and feed intake.

10.7717/peerj.10308/supp-3 Supplemental Information 3 Carcass traits.

10.7717/peerj.10308/supp-4 Supplemental Information 4 Intestinal morphological index.

10.7717/peerj.10308/supp-5 Supplemental Information 5 Intestinal weight and length.

10.7717/peerj.10308/supp-6 Supplemental Information 6 Meat quality.

## Additional Information and Declarations

### Data Availability

The following information was supplied regarding data availability: Raw data are available in the Supplemental Files.
